# Childhood Diarrhoea in the Eastern Mediterranean Region with Special Emphasis on Non-Typhoidal Salmonella at the Human–Food Interface

**DOI:** 10.3390/pathogens8020060

**Published:** 2019-05-06

**Authors:** Ali Harb, Mark O’Dea, Sam Abraham, Ihab Habib

**Affiliations:** 1 College of Science, Health, Education and Engineering, Murdoch University, Perth 6150, Australia; aliharb8@yahoo.com (A.H.); m.o’dea@murdoch.edu.au (M.O.); s.abraham@murdoch.edu.au (S.A.); 2 Thi-Qar Public Health Division, Ministry of Health, Thi-Qar 64007, Iraq; 3 High Institute of Public Health, Alexandria University, Alexandria 21516, Egypt

**Keywords:** Eastern Mediterranean region, non-typhoidal *Salmonella*, zoonoses, child diarrhoea, enteropathogens

## Abstract

Diarrhoeal disease is still one of the most challenging issues for health in many countries across the Eastern Mediterranean region (EMR), with infectious diarrhoea being an important cause of morbidity and mortality, especially in children under five years of age. However, the understanding of the aetiological spectrum and the burden of enteric pathogens involved in diarrhoeal disease in the EMR is incomplete. Non-typhoidal *Salmonella* (NTS), the focus of this review, is one of the most frequently reported bacterial aetiologies in diarrhoeal disease in the EMR. Strains of NTS with resistance to antimicrobial drugs are increasingly reported in both developed and developing countries. In the EMR, it is now widely accepted that many such resistant strains are zoonotic in origin and acquire their resistance in the food-animal host before onward transmission to humans through the food chain. Here, we review epidemiological and microbiological aspects of diarrhoeal diseases among children in the EMR, with emphasis on the implication and burden of NTS. We collate evidence from studies across the EMR on the zoonotic exposure and antimicrobial resistance in NTS at the interface between human and foods of animal origin. This review adds to our understanding of the global epidemiology of *Salmonella* with emphasis on the current situation in the EMR.

## 1. Background

Worldwide, diarrhoeal diseases accounted for 8% of all deaths in children under five years of age in 2016, and this translates to over 1300 young children dying each day or approximately 480,000 children a year [1]. The incidence of diarrhoeal infections among children in the Eastern Mediterranean region (EMR) continues to pose a significant public health challenge in countries across the region. For the purpose of this review, we utilized the regional classification set by the World Health Organization (WHO), and as such, the EMR encompasses the Islamic Republic of Afghanistan (Afghanistan), Djibouti, Somalia, Republic of Yemen (Yemen), Arab Republic of Egypt (Egypt), Islamic Republic of Iran (Iran), Iraq, Jordan, Lebanon, Libya, Morocco, Pakistan, Palestine, Sudan, Syrian Arab Republic (Syria), Tunisia Bahrain, Kuwait, Oman, Qatar, Saudi Arabia, and the United Arab Emirates (UAE). The proportion of paediatric diarrhoea cases has increased over time in several countries in the EMR, as in Iraq—from 14.9% in 1997 to 21.3% in 2000 [2,3]—and Iran, from 10.3% to 19.6% between 2008 and 2010 [4,5]. In Egypt, the incidence of diarrhoea in children has declined from 44% to 23.6% based on reports between 1999 and 2005 [6,7]. Diarrhoea attributed disability-adjusted life years (DALYs) among children under five years of age in the EMR regions were estimated to be 6,058,681 (4,045,101–8,618,353) [8]. Across the 22 countries in the EMR, the highest rates of diarrhoea-attributed mortality among children younger than five years were reported in Somalia, Afghanistan, Djibouti, Iraq, Syria, Yemen, Sudan, Egypt and Tunisia [8,9].

Several countries in the EMR suffer from fragile health care systems, of which Iraq is an example. Several generations of Iraqi children born since the early 1990s have faced adverse conditions negatively impacting their nutrition and health as a result of decades of wars, sanctions and political instability [10]. Diarrhoeal disease is a leading cause of morbidity and mortality among Iraqi children younger than five years [10,11]. The period between 1994 and 1999 witnessed the highest rate of diarrhoea-attributed deaths in Iraq, and in the EMR as a whole, as diarrhoea was a common cause of death in children under five years old; it was responsible for 43.4% of deaths in children aged 2–5 years [11]. Additionally, the 2004 survey of the United Nations International Children’s Emergency Fund (UNICEF) in partnership with the Government of Iraq indicated that approximately 90% of children under the age of five years visited hospitals due to diarrhoea [12]. In this former survey, diarrhoea and acute respiratory infection accounted for 70% of childhood deaths, but the fatality rate due to diarrhoeal illnesses was higher than those caused by respiratory infection [12].

## 2. Epidemiological Aspects of Diarrhoeal Diseases Among Children in the EMR

The majority of diarrhoea infections in children occur during the summer months in countries with a hot and dry climate [13,14]. It has been noted that enteric illnesses in temperate latitudes have a seasonal pattern, with the highest incidence of diseases during the summer months [15]. This is consistent with published evidence of a positive correlation between gastrointestinal infection with enteric pathogens and the increase in ambient temperature [16,17]. Several epidemiological studies confirm the role of age and immune response as important triggers to infectious diarrhoea in children [18,19]. Children below five years of age are significantly more susceptible to diarrhoeal illnesses compared with other age groups [14,18]. In Iraq, a study by Siziya and colleagues (2009) of the prevalence of diarrhoea in 14,676 children less than five years of age revealed that 21.3% of the children had diarrhoea in the two weeks preceding the survey. Based on the aforementioned survey, a history of diarrhoea was positively associated with lower socioeconomic status and a lack of access to clean sources of water [2]. A hospital-based study in Iraq reported a prevalence of diarrhoea in 63.5% of children at three government referral paediatric hospitals in Baghdad [20]. The authors also suggested that this high prevalence rate is likely due to economic collapse, poor sanitation, lack of safe water and inadequate provision of health care [20].

Paediatric diarrhoea has an important financial and productivity impact on the livelihood of families in different countries across the EMR [14,21]. In the United Arab Emirates, Howidi et al. [22] estimated an average cost of $64 for expenses spent dealing with medical care per diarrhoeal episode in children. In Oman, a study by Al Awaidy et al. [23] revealed that the total cost of hospitalization due to diarrhoea (direct medical costs) was estimated at $539 per child for three hospital days, totalling $1.8 million per year for all outpatient and hospital settings in the country. 

## 3. Microbiological Aspects of Diarrhoeal Diseases Among Children in the EMR

The prevalence of enteropathogens in child diarrhoeal illnesses throughout the EMR is difficult to precisely assess due to variations in geographical settings, a lack of harmonization in sampling approaches and study designs and varying laboratory techniques and methods used across different studies, even within the same country [24,25]. Table 1 provides a descriptive summary of various studies reporting the occurrence of major etiological agents responsible for paediatric diarrhoea in different countries across the region. Data on enteric pathogens implicated in diarrhoea among children in the EMR are still limited.

In Iraq, little is known about the causative agents of diarrhoea in children. However, a prospective hospital-based study has shown that *Entamoeba histolytica* is responsible for approximately 85% of diarrhoea infections, and the same study also reported that non-typhoidal *Salmonella* spp. and *Shigella* were isolated from 42% of cases in children under five years of age [26]. In Saudi Arabia, cross-sectional studies have investigated the prevalence of pathogen-induced diarrhoea in faecal samples of children from hospitals and outpatient clinics in different localities. Among the different enteric pathogens found in these studies, rotavirus, *Salmonella* and *Giardia lamblia* were the most prevalent [27,28,29]. Studies conducted in Bahrain [30], Kuwait [31] and Oman [32] (Table 1) shared some common findings, with rotavirus and adenovirus found to be the major viral causes and *Salmonella* and *Shigella* found to be the most common bacterial causes involved in cases of child diarrhoea [31,32]. The authors also observed that symptoms associated with bacterial gastroenteritis were more severe compared to those of a viral nature. In Qatar, noroviruses have been implicated as the predominant viral pathogen associated with severe diarrhoea in children [33]. Microbiological studies on bacterial diarrhoeal illness among hospitalized children in Pakistan [34], Egypt [35,36], Iran [24], Palestine [37], Djibouti [38] and Somalia [39] reported that the main etiologic agents were *Campylobacter*, *Salmonella*, *E. coli* and *Shigella*. Clinical findings in these studies varied according to the aetiology of diarrhoea; however, abdominal pain, vomiting, fever and dehydration were seen in a majority of cases, and the highest incidence rates were commonly reported in the summer months. 

In Libya [40], Sudan [25] and Tunisia [41], the molecular screening of the aetiology of acute diarrhoea among young children has revealed that the major viral agents identified were rotavirus and norovirus, the most frequently diagnosed bacterial pathogens were *Salmonella* spp. and *E. coli* and the most commonly detected parasites were *Giardia lamblia* and *Entamoeba histolytica*. Overall, the above reported studies across different countries in the EMR (Table 1) suggest that knowledge of the aetiology of diarrhoea is important for guiding future epidemiological surveillance and for the implementation of evidence-based public health measures to prevent and control this disease syndrome. *Salmonella* has been featured as one of the leading bacterial causes commonly detected in child diarrhoeal cases across the EMR. In the following section of this review, we will elucidate the state of epidemiological and microbiological features of non-typhoidal *Salmonella* (NTS) implicated in acute paediatric gastroenteritis in children in this region.

## 4. Non-Typhoidal *Salmonella* (NTS)—The Pathogen, Exposure and Illness

As of 2012, t more than 2587 serovars of *Salmonella enterica* have been reported from all over the world, and almost all are able to cause illness in animals and humans including gastroenteritis and other acute infections [44]. *Salmonella* spp. are capable of adapting, growing and/or surviving in a various range of environments including temperatures as high as 54 °C or as low as 2 °C, extracellular pH levels below 3.9 and up to 9.5 and salt concentrations up to 4% wv−1 NaCl [45,46,47]. Such exceptional characteristics can have a significant effect on the survival of *Salmonella* outside of the host organism and in food during processing, preparation, and storage [45,48]. In pure cultures, *Salmonella* spp. are normally inactivated by frozen storage at −22 °C in as few as 5 days [49]; however, freezing does not eliminate the pathogen from contaminated foods [50]. In addition to its survival in extreme conditions, the growth of this pathogen in non-host environments such as natural waters, wastewater sludge, soil and compost has also been reported in several studies [51,52].

There are two major clinical syndromes caused by *Salmonella* infection in human: the first is typhoid and paratyphoid fever, caused by *S.* Typhi and Paratyphi, which are highly adapted to the human host; and the second major clinical syndrome is the gastrointestinal disease caused by a large number of NTS serovars, which are predominantly found in animal reservoirs [53]. The most common mode of NTS infection in human is the ingestion of contaminated food or water [54,55]. Initial symptoms are characterized by an acute onset of fever and chills, nausea and vomiting, abdominal cramping and diarrhoea, and other nonspecific complaints including headache, myalgias, and arthralgias [56,57]. Gastroenteritis caused by NTS is usually self-limiting, lasting for 10 days or less, and may be grossly bloody [54]. *Salmonella* is excreted in faeces after infection, a process that may last for a median of 5 weeks; however, the excretion may be prolonged in young children [58,59]. In rare cases, NTS infections could develop atypical clinical syndromes of variable severity, including bacteraemia, endovascular infection and focal infection [56,60]. In developing countries, children with bacteraemia are more likely to have predisposing conditions, a higher risk for the incidence of meningitis and increased fatality rates compared to adults [58,61]. 

## 5. Implication of NTS in Diarrhoeal Illnesses in the EMR

The global burden of NTS gastroenteritis is estimated to be 93.8 million human infections, with 155 thousand deaths and an average incidence rate of 1.14 episodes/100,000 persons [62]. This reflects the enormous burden of the disease in both industrialized and developing countries [63]. For the WHO-defined EMR, the median incidence rate of NTS is 1610 illnesses, with 0.6 deaths per 100,000 persons [64]. The incidence rate of salmonellosis varies substantially between countries across the EMR and is influenced largely by the absence of systematic, harmonized national and regional surveillance and reporting systems. An epidemiological survey in Qatar spanning eight years (2004 to 2012) reported that the incidence rate of reported NTS associated illnesses ranged between 12.3 and 18.1 cases per 100,000 inhabitants, with most reported NTS cases occurring in children under the age of five [65]. In Lebanon, the Department of Epidemiologic Surveillance data from 2001 to 2013 indicated that the annual incidence rate of reported salmonellosis was 13.3 per 100,000 individuals, with an increasing trend of the number of NTS cases between 2009 and 2013 [66]. In Jordan, the reported rate of human salmonellosis is alarmingly higher than what is reported elsewhere across the EMR, with a notification rate of 124 cases per 100,000 persons, as reported in a study from 2003 to 2004 [67]. Significant associations between climatic factors and NST infections have been reported in Iraq [68], Jordan [42], Tunisia [69], Iran [24], Saudi Arabia [70] and Qatar [33]. It has been documented that ambient temperatures contribute directly to *Salmonella* multiplication in foods, water and contaminated environments and thus propagate the likelihood of infection [15,71].

Few studies have been carried out to elucidate the epidemiology of gastrointestinal salmonellosis in the EMR, particularly on children. The prevalence rates of NTS range from as low as 0.2% to as high as 34% [27,39], with the highest reported age-related prevalence usually among children under the age of five (Table 2). Published studies reporting the rates of NTS in the EMR countries are summarised in Table 2. Studies from Iraq (Mosul) [68], Iran (Tehran) [72], Saudi Arabia [27], Kuwait [31], Morocco (Marrakesh) [43] and Yemen [73] reveal a noteworthy high incidence rate (15% to 34%) of NTS (Table 2). 

Several published studies (Table 2) indicate that the most widely reported serovars associated with acute diarrhoeal disease across the EMR are the *Salmonella* serovar Typhimurium and *Salmonella* serovar Enteritidis [73,76,80]. Similar to the situation in EMR, *S.* Typhimurium followed by *S*. Enteritidis are also the top-ranked serovars involved in human diarrhoeal illnesses across Africa, North America and Oceania (Australia and New Zealand) [82]. In contrast, *S.* Enteritidis is more frequently reported than *S.* Typhimurium in human clinical isolates in Europe, Asia and Latin America [83].

The difference in *Salmonella* prevalence and the diversity of NTS serovars among humans is dynamic in nature, and it is not surprising to capture variations between countries. Such variations might be attributed to several factors impacting NTS levels in food and water, which play a major role in human exposure to infection [84]. Among these factors are climate, food-animal production practices, the level of spread of specific serotypes in environmental reservoirs and the availability of vaccination programs in food animals. Eggs and poultry products have been described as the main vehicles for the transmission of human salmonellosis, accounting for the majority of foodborne outbreaks [84,85].

## 6. NTS and the Risk of Zoonotic Exposure from Chicken Meat Products

Several studies in the EMR investigated the prevalence of NTS in local and imported chicken meat, as summarised in Table 3. Rates of *Salmonella* contamination vary between countries due to a number of factors including the source and type of sample, slaughterhouse sanitation, the level of cross-contamination at the retail level and the detection methods employed [76,86]. In Iraq, *Salmonella* contamination was reported in 26% of fresh retail chicken meat samples [87,88] and in 39% of raw and frozen chicken carcasses [89]. Studies in several EMR countries have identified low *Salmonella* prevalence rates in chicken meat, such as in Kuwait [90], Tunis/Tunisia [91], Saudi Arabia/Riyadh [92], and Egypt [93,94]. Several studies indicated that *S*. Typhimurium and *S*. Enteritidis were the most prevalent serovars in chicken meat in the EMR [86,93,95,96]. Interestingly, the two most commonly reported *Salmonella* serovars in human diarrhoeal illness are also the two commonly recovered serovars from chicken meat, highlighting the role of chicken meat as an important source of salmonellosis in this region.

To study the zoonotic transmission of NTS at the human–animal–food interface, it is important to employ the advances in molecular epidemiology tools. Pulsed-field gel electrophoresis (PFGE) and multiple-locus variable-number tandem repeat analysis (MLVA) have been widely considered to be the gold standard molecular subtyping and fingerprinting methods for tracking the source of *Salmonella* contamination [108,109]. However, in recent years, Whole Genome Sequencing (WGS) has become a powerful tool in elucidating transmission pathways [110]. For *Salmonella*, WGS provides a massive amount of data for research purposes along with the rapid acquisition of multilocus sequence types (MLST), serotypes and antimicrobial resistance gene data [111,112,113]. Sequence-based typing can also be used to obtain basic biological insights to explain the associations between isolates, thus providing added value to the source attribution [112,113,114]. In addition to its high discrimination ability, WGS could provide additional data about virulence determinants and genome evolution, and such results can be easily shared and communicated [115,116,117].

## 7. Antimicrobial Resistance in NTS at the Interface between Human and Food of Animal Origin in the EMR

Antimicrobial therapy is recommended in severe cases and/or cases of prolonged enteritis, meningitis, septicaemia and extra-intestinal complications associated with salmonellosis [53,58,60]. Antimicrobial resistance in NTS has increased in recent years worldwide, due to the widespread use of antimicrobial drugs in human and veterinary sectors, and poses an on-going threat to global public health [118,119,120,121]. The incidence of resistance to traditional antibiotics (e.g., ampicillin, tetracycline and streptomycin) is evident to be high in *Salmonella* isolates from foods of animal origin, especially poultry, in EMR countries [86,87,98,99,100,101,102,103,104,105,106,107,108,109,110,111,112,113,114,115,116,117,118,119,120]. This finding is highly concerning from a public health perspective, as many of these traditional (1st generation) antibiotics are still widely prescribed to treat diarrhoea in children and adults due to their low cost and availability in developing countries, including countries of the EMR [121,122]. Similar patterns of high resistance to these traditional antibiotics are also evident in *Salmonella* isolated from human enteric illnesses, especially in Iraq [68], Kuwait [31], Saudi Arabia [123], Jordan [77], Iran [76], Oman [124] and Libya [40].

Studies in Saudi Arabia [123], and Kuwait [125] reported frequent resistance to chloramphenicol in NTS isolated from childhood diarrhoea (although it is not approved for human use). Resistance to chloramphenicol in *Salmonella* is facilitated by type *A* chloramphenicol acetyltransferase genes (*catA1* and *catA2*) or by cassette-borne type B chloramphenicol acetyltransferase genes (*catB2*, *catB3* or *catB8*) [126,127]. Furthermore, two new chloramphenicol/florfenicol exporter genes, *cmlA9* and *floR*, have recently been identified for phenicol resistance genes in *Salmonella* isolates [121,122,123,124,125,126,127,128].

Resistance to nalidixic acid (NA) in *Salmonella* isolates from paediatric cases with enteritis was as high as 84.2% in a study in Libya [40] and was detected at a rate of 42.3% in a study in Iran [72]. There is an alarming concern over the increase in the resistance of NTS to ciprofloxacin and extended-spectrum cephalosporins [118,129], given the critical clinical relevance of these antimicrobials. Chromosomal mutations in the genes encoding topoisomerase II, *gyrA* and *gyrB*, and/or topoisomerase IV, *parC* and *parE*, accounting for resistance to quinolones/fluoroquinolones, are known to occur in *Salmonella* isolates [130]. More recently, various plasmid-mediated quinolone resistance (PMQR) genes including genes *qnrD*, *qnrA*, *qnrB* and *qnrS* variants, all of which code for DNA topoisomerase protecting proteins, as well as the genes *qepA* and *qepAB* coding for a quinolone-specific efflux pump, and the acetyltransferase *aac(60)-Ib-cr,* have been identified in *Salmonella* isolates [131,132,133]. In some EMR countries, the increase in resistance is rapid and considerable; in Libya, a study reported that 63.1% of human *Salmonella* isolates were ciprofloxacin-resistant [40]. 

Resistance to β-lactam antibiotics (penicillins, cephalosporins, and carbapenems) in *Salmonella* is mediated by a wide range of β-lactamase enzymes [134]. To date, genes coding for 13 different types of β-lactamases have been known in *Salmonella*. Among them, *bla_AAC_*/*bla_DHA_*/*bla_CMY_*/*bla_TEM_* genes are of particular importance as the first representative encoding of cephalosporinases that hydrolyse most β-lactamase except carbapenems [129]. Extended-spectrum cephalosporins are the antimicrobials of choice for invasive *Salmonella* treatment, especially in children where treatment with fluoroquinolones is not recommended [130]. A study by Rotimi et al. [135] observed resistance to cefotaxime and ceftriaxone among *Salmonella* spp. isolated from stool samples of patients with acute diarrhoea and septicaemia during 2003–2005 in Kuwait and United Arab of Emirates.

## 8. Conclusions

This review consolidates recent updates on the spectrum of enteric pathogens in the EMR region, with special emphasis on NTS. Among bacterial pathogens, NTS infections continue to pose a distressing public health concern, notably in children under five years old. The emergence of antimicrobial resistance in *Salmonella* strains present a great challenge at the human–food–environment interface in terms of the effective treatment of the infections caused by these strains. The EMR spans different countries with varying and evolving socio-economic statuses. Several countries in the EMR, notably Iraq, Yemen and Syria, are experiencing similar challenges as a result of fragile political situations and insecurity as well as sanitation, food safety, and food security issues and an influx of refugees. The public health system in several countries in the EMR is struggling to respond to the evolving burden of enteric illnesses due to the lack of surveillance of important enteric pathogens, such as *Salmonella*, at hospital, household and food chain levels and would benefit from a multi-dimensional research approach encompassing these levels. Bacterial infections and their antimicrobial resistance profiles should be monitored more closely across the EMR, especially in vulnerable groups such as children less than five years old. Studies focusing on investigating epidemiological and microbiological aspects of infectious diarrhoea in underprivileged communities/regions at the national level should be prioritized in future research.

## Figures and Tables

**Table 1:** Distribution of different pathogens from diarrhoeal stool samples among children across countries in the Eastern Mediterranean region.

Country/Locality	Study Period/Month	Population/Age	No. of Stool Samples	Prevalence of Enteropathogens			Reference
				Virus (%)	Bacteria (%)	Parasite (%)	
Bahrain	20	Children < 15 years	805	Rotavirus (13.9) Adenovirus (0.6)	*Salmonella* spp. (5.7) *Shigella* spp. (3.2) *Campylobacter jejuni* (1.6) *Enteropathogenic* *Escherichia coli* (*E. coli*) (0.5)	ND (not detected)	[30]
Djibouti	1	Children < 16 years	209	ND	*Enteroadherent E. coli* (EAEC) (10.6) *Enterotoxigenic E. coli* (ETEC) (11.0) *Enteropathogenic E. coli* (EPEC) (7.7) *Shigella* spp. (7.7) *Salmonella* spp. (2.9) *Campylobacter jejuni/coli* (3.3) *Aeromonas hydrophila* (3.3)	ND	[38]
Egypt/Alexandria	Not described	Children mean age 9.8 months	880	ND	*Campylobacter* spp. (17.2) *Salmonella* spp. (3) *Shigella* spp. (2)	ND	[35]
Egypt/Fayoum	2	Children < 5 years	356	Rotavirus (17)	*Enterotoxigenic E. coli* (ETEC) (10.8) *Campylobacter* spp. (5.6) *Shigella* spp. (2.0) *Salmonella* spp. (0.6) *Aeromonas hydrophila* (1.1) *Vibrio fluvialis* (0.6)	*Cryptosporidium* (10.7)	[36]
Iran/Tehran	24	Children < 5 years	1078	ND	*Shigella* spp (26.7) *Shiga-like toxin producing E. coli* (STEC) (18.9) *Enteroaggregative E. coli* (EAEC) (16.6) *Enteropathogenic E. coli* (EPEC) (12.6) *Campylobacter* spp. (10.8) *Salmonella* spp. (7.6) *Enterotoxigenic E. coli* (ETEC) (6.8)	ND	[24]
Iraq/Baghdad	Not described	Children < 10 years	1500	ND	*Salmonella* spp. (4.28) *Shigella* spp. (2.14)	*Entamoeba histolytica* (83.58)	[28]
Jordan/Irbid	12	Children < 12 years	265	Rotavirus (32.5)	*Enteropathogenic E. coli* (12.8) *Enteroaggregative E. coli* (10.2) *Enterotoxigenic E. coli* (5.7) *Shigella* spp. (4.9) *Salmonella* spp. (4.5) *Campylobacter jejuni/coli* (1.5) *Enteroinvasive E. coli* (1.5)	*Entamoeba histolytica* (4.9) *Cryptosporidium* spp. (1.5) *Giardia lamblia* (0.8)	[42]
Kuwait	15	Children (not described)	621	Rotavirus (45.0) Adenovirus (4.0)	*Salmonella* spp. (24) *Enterotoxigenic E. coli* (9) *Campylobacter jejuni* (7) *Enteropathogenic E. coli* (7) *Shigella* (4)	ND	[31]
Libya/Tripoli	8	Children < 5 years	239	Norovirus (15.5) Rotavirus (13.4) Adenovirus (7.1) Astrovirus (1.7)	*E. coli* (11.2) *Salmonella* spp. (9.7) *Shigella* spp. (0.8) *Campylobacter* spp. (2.9) *Aeromonas* spp. (4.2) *Cryptosporidium* spp. (2.1)	*Entamoeba histolytica* (0.8) *Giardia lamblia* (1.3)	[40]
Morocco/Rabat	13	Children < 5 years	122	Rotavirus (17.2) Astrovirus (4.9) Hepatitis A (0.8) Norovirus (0.8)	*E. coli* (58.2) *Shigella* spp. (7.4) *Salmonella* spp. (4.1) *Campylobacter* spp. (4.1)	*Giardia intestinalis* (0.8) *Entamoeba histolytica* (0.8)	[43]
Oman/Muscat	24	Children < 5 years	217	Rotavirus (31.0) Adenovirus (4.0)	*E. coli* (10) *Shigella* (7) *Campylobacler* spp. (2) *Salmonella* spp. (2)	*Giardia lamblia* (11), *Entamoeba histolytica* (9)	[32]
Pakistan/Karachi and Rawalpindi	24	Children < 3 years	402	Rotavirus (8.2)	*Enteropathogenic E. coli* (EPEC) (32.8) *Enterotoxigenic E. coli* (ETEC) (14.2) *Shigella* spp. (3.2) *Salmonella* spp. (2)	ND	[34]
Palestine/Gaza	12	Children < 12 years	300	ND	*Enterohemorrhagic E. coli* (8.3) *Shigella* spp. (6.7) *Campylobacter jejuni* (5) *Salmonella* spp. (4) *Yersinia enterocolitica* (2.7) *Aeromonas* spp. (4.7) *Plesiomonas* spp. (1.3)	ND	[37]
Qatar	6	Children (not described)	288	Norovirus (28.5) Rotavirus (10.4) Adenovirus (6.25) Astrovirus (0.30)	*Salmonella* spp. (8) *Escherichia coli* (3) *Shigella* spp. (1.5) *Campylobacter* spp. (1.5)	ND	[33]
Saudi Arabia/Eastern Province	19	Children (not described)	853	Rotavirus (11.5)	*Salmonella* spp. (34) *Shigella* spp. (14.7)	*Entamoeba histolytica* (13.5) *Giardia intestinalis* (10.4)	[27]
Saudi Arabia/Jeddah	12	Children < 5 years	576	Rotavirus (34.6)	*E. coli* (13) *Klebsiella pneumoniae* (4) *Salmonella* spp. (3) *Shigella flexneri* (2.6)	*Giardia lamblia* (3.1) *Entamoeba histolytica* (2.2) *Trichuris trichiura* (0.7) *Hymenolepis nana* (0.7) *Ascaris lumbricoides* (0.7)	[28]
Saudi Arabia/Najran region	9	Children < 5 years	326	Rotavirus (17.2) Adenovirus (3.7) Astrovirus (1.2)	*Salmonella* spp. (8.6) *Shigella* spp. (2.1)	*Giardia lamblia* (0.9) *Entamoeba histolytica* (0.3)	[29]
Somalia Mogadishu	12	Children < 14 years	1667	Rotavirus (25)	*Enterotoxigenic E. coli* (ETEC) (11) *Shigella* spp. (9) *Campylobacter jejuni* (8) *Vibrio cholerae non-O1* (6) *Salmonella* spp. (4) *Aeromonas hydrophila* (9) *Plehnwnas shigelloides* (2)	*Giardia intestinalis* (8) *Entamoeba histolytica* (2)	[39]
Sudan/Khartoum	12	Children < 5 years	437	Rotavirus A (22)	*Enteroaggregative E. coli* (EAEC) (21) *Enteropathogenic E. coli* (EPEC) (14) *Enterotoxigenic E. coli* (ETEC) (9) *Enteroinvasive E. coli* (EIEC) (4) *Shigella sonnei* (3) *Shigella flexneri* (4) *Shigella dysenteriae* (1) *Salmonella typhi* (2) *Salmonella paratyphi C* (1) *Campylobacter jejuni* (3)	*Giardia intestinalis* (11), *Entamoeba histolytica* (5)	[25]
Tunisia	12	Children < 5 years	124	Rotavirus (33.9) Norovirus (8.9)	*Enteroaggregative E. coli* (EAEC) (23.4) *Enteroinvasive E. coli* (EIEC) (12.1) *Enteropathogenic E. coli* (EPEC) (13.7) *Enterotoxigenic E. coli* (ETEC) (21) *Enterohemoragic E. coli* (EHEC) (1.6) *Salmonella* spp. (9.7)	*Entamoeba coli* (1.6) *Cryptosporidies* (1.6) *Giardia lamblia* (0.8) *Blastocystis hominis* (0.8)	[41]

**Table 2:** Prevalence of *Salmonella* detected in children with acute diarrhoea in the Eastern Mediterranean region.

Country/Locality	Study Period/Month	Population/Age	Source of Cases	Methods	Number of Acute Diarrhoeal Cases	Prevalence of *Salmonella* Infected (%)	Predominate S. Serovar	References
Bahrain	24	Children < 15 years	Hospital admissions	*Salmonella* culture (stool)	805	46 (5.7)	Typhimurium	[30]
Djibouti	1	Children < 16 years	Health centres	*Salmonella* culture (stool)	209	6 (2.9)	NS (not specified)	[38]
Egypt/Aswan	Not described	Children (not described)	Outpatients and inpatients	*Salmonella* culture (stool)	151	11 (7)	NS	[74]
Egypt/Cairo	Not described	Children < 5 years	Hospital admissions	*Salmonella* culture (stool)	356	5 (1.4)	NS	[75]
Egypt/Fayoum	2	Children < 5 years	Hospital admissions	*Salmonella* culture (stool)	356	*2* (0.6)		[36]
Iran	36	Children < 5 years	Children hospitals admissions	*Salmonella* culture and PCR	555	42 (7.6)	NS	[24]
Iran/Tehran	108	Children < 14 years	Hospital admissions	*Salmonella* culture (stool)	2487	700 (28.14)	NS	[72]
Iran/Tehran	24	Children < 12 years	Paediatric hospital admissions	*Salmonella* culture (stool)	5900	139 (2.4)	Typhimurium Enteritidis	[76]
Iraq/Mosul	Not described	Children < 7 years	Paediatric hospital admissions	*Salmonella* culture (stool)	111	17 (15)	Typhimurium Worthington	[68]
Iraq/Baghdad	Not described	Children < 10 years	Paediatric hospital admissions	*Salmonella* culture (stool)	420	18 (4.7)	NS	[26]
Jordan/Irbid	12	Children < 12 years	Hospitalized children	*Salmonella* culture (stool)	265	12 (4.5)	NS	[42]
Jordan	48	Children (Not described)	Hospital admissions	*Salmonella* culture (stool)	1400	150 (10.7)	NS	[77]
Kuwait	15	Children (Not described)	Hospitalized children	*Salmonella* culture (stool)	621	149 (24)	NS	[31]
Libya/Zliten	12	Children < 12 years	Hospital admissions	*Salmonella* culture (stool)	169	23 (13.6)	Heidelberg Enteritidis	[78]
Morocco/Marrakesh	12	Children < 15 years	Patient children in household	*Salmonella* culture (stool)	390	127 (32·56)	*Salmonella* group A *Salmonella* group B *Salmonella* group C *Salmonella* group D	[79]
Morocco/Rabat	13	Children < 5 years	Hospital admissions	*Salmonella* culture (stool)	122	5 (4.1)	NS	[43]
Oman/Muscat	24	Children < 5 years	Hospital admissions	*Salmonella* culture (stool)	217	5 (2)	NS	[32]
Pakistan/Karachi and Rawalpindi	24	Children < 3 years	Hospital admissions	*Salmonella* culture (stool)	402	8 (2)	NS	[34]
Palestine/Gaza	12	Children < 12 years	Hospital admissions	*Salmonella* culture (stool)	300	12 (4)	NS	[37]
Qatar	5	Children (Not described)	Hospital admissions	*Salmonella* culture (stool)	288	23 (8)	NS	[33]
Saudi Arabia/Yanbu	4	Children (Not described)	Hospital admissions	*Salmonella* culture (stool)	136	15 (11)	Typhimurium Enteritidis Virchow	[80]
Saudi Arabia/Eastern Province	19	Children (Not described)	Hospital admissions	*Salmonella* culture (stool)	853	290 (34)	NS	[27]
Saudi Arabia/South Jeddah	12	Children < 16 years	Hospital admissions	*Salmonella* culture (stool)	367	56 (15.3)	NS	[70]
Somalia/Mogadishu	12	Children < 14 years	Hospital admissions	*Salmonella* culture (stool)	1667	4 (0.2)	NS	[39]
Sudan/Khartoum	12	Children < 5 years	Children in rural area	*Salmonella* culture (stool)	437	3 (0.7)	Enteritidis	[81]
Tunisia/Tunis	48	Children 1–15 years	Paediatric and health centres	*Salmonella* culture (stool)	115	11 (9.5)	Enteritidis Anatum	[69]
Yemen/Thamar	12	Children (Not described)	Hospital admissions	*Salmonella* culture (stool, blood, urine)	460	73 (15.9)	Typhimurium Enteritidis	[73]

**Table 3:** Prevalence of *Salmonella* detected in chicken meat in the Eastern Mediterranean region.

Country/Locality	Source of Samples	No. Positive/No. Test (% Prevalence)	Methods	Predominant *S.* Serovars	Reference
Egypt/Dakahlia, Gharbia, Damietta and Kafr el-Sheikh	Chicken meat	14/320 (5)	*Salmonella* culture and PCR	Typhimurium Enteritidis Infantis	[93]
Egypt/Mansoura	Chicken carcasses Chicken drumsticks Chicken gizzards Chicken livers	8/50 (16) 14/50 (28) 16/50 (32) 30/50 (60)	*Salmonella* culture and PCR	Enteritidis Typhimurium Kentucky	[86]
Egypt/Minoufiya and Cairo	Chicken meat	6/100 (6)	*Salmonella* culture and matrix-assisted laser desorption/ionization time-of-flight (MALDI-TOF)	Typhimurium Enteritidis	[94]
Egypt/Zagazig	Chicken meat	7/50 (14)	*Salmonella* culture and PCR	Typhimurium	[97]
Iran/Alborz	Chicken meat Chicken liver Chicken heart Chicken gizzard	58/200 (29) 26/120 (21.6) 17/120 (14.1) 10/120 (8.3)	*Salmonella* culture	Thompson Enteritidis Typhimurium	[87]
Iran/Tehran	Chicken meat	86/190 (45)	*Salmonella* culture	Thompson Haardt Enteritidis	[98]
Iraq/Baghdad	Chicken meat	39/100 (39)	*Salmonella* culture	Infantis Enteritidis Vichow	[89]
Iraq/Mosul	Chicken meat	21/81(26)	*Salmonella* culture	Infantis Zanzibar Anatum	[88]
Jordan/Irbid	Chicken meat	91/104 (87.5)	*Salmonella* culture and PCR	NS	[99]
Jordan/Northern	Chicken product (shawarma)	30/80 (37.5)	*Salmonella* culture and PCR	Paratyphi A Cholerasuis Pullorum	[100]
Jordan/Northern	Chicken meat	200/302 (66)	*Salmonella* culture and PCR	Enteritidis Cholerasuis,	[101]
Kuwait	Chicken carcasses	11/180 (6.1)	*Salmonella* culture	Enteritidis Infantis	[90]
Libya/Tripoli	Chicken product (burgers)	15/120 (12.9)	*Salmonella* culture	NS	[102]
Morocco/Tetouan	Chicken meat	18/86 (20.9)	*Salmonella* culture	Kentucky Typhimurium, Enteritidis Agona	[95]
Pakistan/Hyderabad	Chicken meat	38/100 (38)	*Salmonella* culture	Enteritidis Typhimurium.	[103]
Pakistan/Karachi	Chicken carcasses	78/160 (48.75)	*Salmonella* culture	Enteritidis Typhimurium	[96]
Saudi Arabia/Riyadh	Chicken liver Chicken heart Chicken spleen	209/3284 (6.3) 107/2315 (4.6) 45/899 (5.0)	*Salmonella* culture	Enteritidis Virchow	[92]
Sudan/Khartoum	Chicken meat	35/193 (18.13)	*Salmonella* culture	Stanleyville Kentucky Virchow Hadar Typhimurium	[104]
Syria	Chicken meat	32/100 (32)	*Salmonella* culture	NS	[105]
Tunis	Chicken meat	29/60 (48.3)	*Salmonella* culture and PCR	Typhimurium Zanzibar Orion	[106]
Tunis/Tunisia	Chicken meat	29/433 (6.7)	*Salmonella* culture and PCR	Kentucky Anatum Zanzibar	[91]
United Arab Emirates/Dubai	Chicken meat	28/60 (46.67)	*Salmonella* culture	NS	[107]
